# Identifying Conservation and Conflict Zones for Tibetan Brown Bears Under Climate Change Through Integrated Habitat and Prey Modeling on the Qinghai‐Tibet Plateau

**DOI:** 10.1002/ece3.72941

**Published:** 2026-01-12

**Authors:** Qiaoyun Sun, Kunyuan Wanghe, Yunchuan Dai

**Affiliations:** ^1^ School of Architecture and Urban Planning Shenzhen University Shenzhen China; ^2^ Qinghai Provincial Key Laboratory of Animal Ecological Genomics, Northwest Institute of Plateau Biology Chinese Academy of Science Xining China; ^3^ Institute for Ecology and Environmental Resources Chongqing Academy of Social Sciences Chongqing China

**Keywords:** climate change, habitat vulnerability, human‐bear conflicts, prey distribution, *Ursus arctos pruinosus*

## Abstract

As climate change accelerates ecosystem transformation across high‐altitude landscapes, understanding the shifting dynamics of predator–prey interactions becomes increasingly critical for conserving apex carnivores. To evaluate the spatiotemporal dynamics of potential habitats for the Tibetan brown bear ( *Ursus arctos pruinosus* ) under future climate change scenarios, our study integrates the distribution patterns of its primary natural prey across the Qinghai‐Tibet Plateau. We aim to identify suitable habitats and potential human‐bear conflict hotspots by coupling predator–prey ecological relationships with environmental drivers, thereby providing a refined understanding of habitat suitability and conservation risk under climate‐induced landscape change. We employed the MaxEnt model combined with multi‐source environmental variables to predict the potential habitats of the brown bear under different climate scenarios. To capture the influence of prey distribution and habitat overlap, three ecological relationship scenarios were designed: (S1) ideal distribution range; (S2) stepping stone; and (S3) potential human‐bear conflict area. These scenarios were simulated and compared to examine the influence of prey availability and habitat configuration on brown bear habitat dynamics and conflict vulnerability under climate change. We found that, according to model projections under the RCP4.5 scenario, suitable habitat for the Tibetan brown bear is expected to decline by 16.78%, with core habitats contracting and shifting toward central and western Xizang and southern Qinghai. Marmots showed stable distributions with centroid shifts, maintaining Qinghai as the core area. In contrast, pikas were highly sensitive to land‐use changes, with potential habitat losses of 44.47% and 89.39% in the plateau margins of Sichuan and Yunnan provinces under the RCP8.5 scenario. S3 is projected to expand by 17.03% under RCP4.5, posing additional conservation challenges. The results highlight growing risks of habitat fragmentation and increased human‐wildlife conflicts. We proposed a regionally coordinated conservation framework centered on “core habitat protection—connectivity enhancement—conflict mitigation” to address these emerging threats under climate change.

## Graphical abstract

This study evaluates the future habitat dynamics of the Tibetan brown bear on the Qinghai‐Tibet Plateau under climate change by integrating predator–prey relationships and environmental variables using the MaxEnt model. Results show significant habitat contraction and shifting patterns, increasing the risk of human‐bear conflict. A targeted conservation strategy is proposed to enhance habitat connectivity and mitigate conflict under changing climatic conditions.

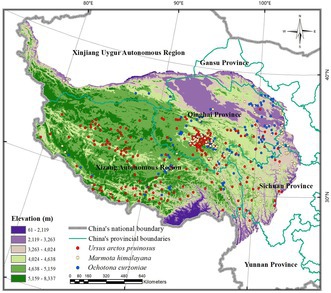

## 1 Introduction

Mountain ecosystems are increasingly recognized as critical yet vulnerable frontiers in the global biodiversity crisis (Theobald et al. 2024). Among these, the Qinghai‐Tibet Plateau (QTP), the world's highest and most expansive alpine ecosystem, plays a pivotal role in understanding ecological responses to environmental change, owing to its exceptional climatic sensitivity, geographic isolation, and relatively intact ecological assemblages (Lu et al. 2017; Wang et al. 2007). However, accelerating climate warming, land‐use intensification, and infrastructure expansion are synergistically reshaping the distributional patterns, phenology, and trophic relationships of native species across the plateau (Wei et al. 2022). These changes are especially concerning for cold‐adapted and range‐restricted taxa, which face heightened extinction risk due to upward habitat shifts, fragmentation, and declining connectivity (Li 2019). Furthermore, as a global climate change sentinel and a vital reservoir of freshwater and endemic biodiversity, the QTP offers a unique opportunity to investigate species responses to rapid environmental transitions in high‐elevation contexts (Miehe et al. 2008; Yao et al. 2012). Consequently, the QTP represents a key region for assessing the ecological vulnerability of endemic species and informing conservation strategies under future global change scenarios.

Large carnivores are widely recognized as keystone species and key indicators of ecosystem integrity due to their expansive home ranges, regulatory roles in trophic dynamics, and high sensitivity to ecological changes (Aryal et al. 2010; Aryal, Hopkins, et al. 2012; Ripple and Beschta 2012). The Tibetan brown bear ( *Ursus arctos pruinosus* ) is an apex omnivore endemic to the Qinghai‐Tibet Plateau (QPT). In China, it is classified as a second‐class national protected species. Understanding of the species' ecological requirements, spatial distribution, and population dynamics remains limited, largely due to its elusive behavior, low population density, and occupation of remote alpine habitats (Dai et al. 2020). Globally, *Ursus arctos* is classified as Least Concern (LC) by the International Union for Conservation of Nature (IUCN), owing to its wide distribution and relatively large population. However, isolated populations, including those in the Himalayas and on the Qinghai‐Tibet Plateau, are exposed to region‐specific threats such as habitat fragmentation, human‐wildlife conflict, and environmental change, underscoring the urgent need for targeted conservation strategies (Aryal et al. 2010; Wu 2014; Dai et al. 2020).

Although brown bears exploit a diverse omnivorous diet that includes over 50 plant species (Nawaz et al. 2019), they primarily rely on animal prey, particularly the Himalayan marmot ( *Marmota himalayana* ) and the plateau pika ( *Ochotona curzoniae* ) (Wu 2014; Xu et al. 2006). These key prey species not only provide essential energy resources but also play functionally important roles in shaping soil processes and grassland dynamics (Smith and Foggin 1999). For instance, their burrowing activities enhance soil aeration and promote plant diversity, while fluctuations in their populations can trigger broader predator–prey cascades within the alpine food web (Smith and Foggin 1999; Zhao et al. 2021). Consequently, the persistence of brown bears is closely linked to both the availability of critical animal prey and the functional integrity of alpine ecosystems.

Despite the ecological significance of the brown bear and its primary prey, research on their projected responses to accelerating climate change and land‐use transformations on the QTP remains insufficient (Dai et al. 2019). Most existing Species Distribution Modeling (SDM) studies have adopted a single‐species framework or focused narrowly on taxonomic subsets, often overlooking the complex ecological interdependencies among predators and prey, as well as the spatial intersection of wildlife habitats with expanding human activity (Guisan and Thuiller 2005). This analytical gap undermines the development of spatially nuanced, ecosystem‐based conservation strategies. In particular, limited attention has been paid to how projected environmental changes may affect the configuration of core habitats, ecological corridors, and zones of elevated human‐wildlife conflicts (Nyhus 2016; Aryal, Raubenheimer, et al. 2012; Ji et al. 2024). Recent field reports and case studies have highlighted a rising frequency of livestock depredation and house break‐ins incidents involving brown bears, particularly in frontier pastoral landscapes of Qinghai Province and Xizang Autonomous Region of China, underscoring the urgency of incorporating human dimensions into species distribution forecasts (Dai et al. 2020). An integrated, multi‐species modeling approach is thus urgently needed to support forward‐looking conservation planning under compounded climate and anthropogenic pressures.

In our study, we assessed the vulnerability of Tibetan brown bear habitat under projected climate change and land‐use scenarios by explicitly incorporating prey species distribution patterns and human disturbance gradients into spatial ecological models. Using the MaxEnt model and multi‐source environmental data, we (1) predicted current and future habitat suitability for the brown bear and its two main prey species under RCP2.6, RCP4.5, and RCP8.5 scenarios; (2) examined the differential contributions of climatic, topographic, and land‐use variables to habitat suitability; and (3) identified potential shifts in habitat networks, focusing on three scenarios: ideal distribution range, stepping stone, and potential human‐bear conflict area. By adopting a comparative modeling framework that jointly considers predator–prey dynamics and landscape heterogeneity, our study provided a spatially explicit basis for formulating regionally coordinated conservation strategies on the QTP, with broader relevance to mountain carnivore conservation under global change. By incorporating species interactions into spatial conservation planning, our work contributes to advancing predictive ecology and informing practical conservation strategies in complex mountain ecosystems.

## 2 Material and Methods

### 2.1 Study Area

**FIGURE 1:**
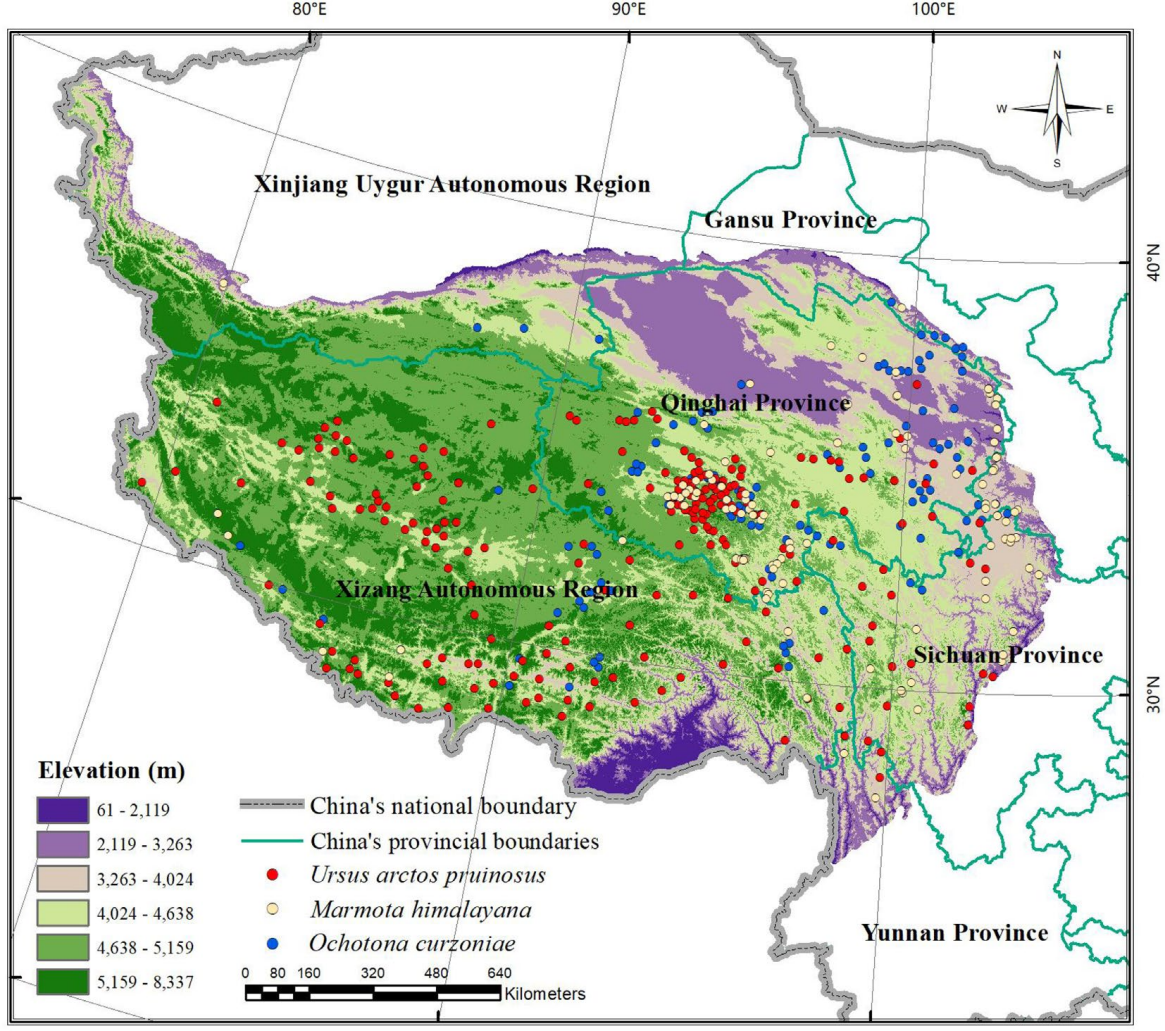
Location of the Qinghai‐Tibet Plateau.

The Qinghai‐Tibet Plateau (QTP; 26°00′–39°47′ N, 73°19′–104°47′ E; Figure 1) is the largest plateau in China and the highest in the world, often referred to as the “Roof of the World” and the “Third Pole.” It extends approximately 2800 km from east to west and spans 300–1500 km from north to south, covering a total area of about 2.5 million square kilometers. The plateau encompasses the entire Xizang Autonomous Region and parts of Qinghai, Xinjiang, Gansu, Sichuan, and Yunnan provinces. The elevation of the QTP generally ranges from 3000 to 5000 m, with an average altitude exceeding 4000 m, making it the source region for many major rivers in East Asia, Southeast Asia, and South Asia. As one of the world's most significant biodiversity hotspots, the QTP harbors an exceptionally rich and unique array of biological diversity, shaped by its distinct geographical environment and complex climatic conditions (Miehe et al. 2010; Myers et al. 2000). The dramatic elevation gradients across the plateau give rise to a variety of ecosystems, including alpine meadows, grasslands, wetlands, lakes, and forests, providing diverse habitats for numerous species. The region is home to a wide range of endemic and flagship species, such as the Tibetan antelope ( *Pantholops hodgsonii* ), snow leopard ( *Panthera uncia* ), Tibetan brown bear, wild yak ( *Bos mutus* ), and black‐necked crane ( *Grus nigricollis* ), which play crucial roles in the ecological stability of the plateau (Schaller 2000).

### 2.2 Collection of Distribution Data for Three Species

Our research team has been dedicated to studying human‐bear conflicts on the QTP, conducting systematic fundamental research on the behavior, habitat, and food resources of brown bears. From 2014 to 2025, our team carried out extensive field investigations on brown bear ecology. During field surveys, we focused on habitat types preferred by brown bears (e.g., alpine meadows and rocky mountains) and foraging sites (e.g., areas near winter houses and garbage dumps), systematically collecting evidence of brown bear presence, including direct sightings, feces, footprints, feeding traces, hair, and dens. Through field surveys and literature reviews, a total of 585 brown bear occurrence records were collected, of which 184 were derived from published literature (Jien and Harris 2007; Su et al. 2018). Additionally, during the field surveys, we simultaneously recorded the occurrence points of its primary prey species. A total of 308 marmot occurrence records were compiled, including 216 field‐based observations (visual sightings and burrow signs) and 92 records extracted from published literature (Wang et al. 2023), which were originally derived from verified field surveys. Similarly, 311 plateau pika occurrence records were collected, comprising 236 field observations and 75 literature‐based records from Qi et al. (2022), which also originated from empirical monitoring data. To reduce spatial autocorrelation and enhance the reliability of the modeling process, a 10 km^2^ grid‐based filtering strategy was employed, where only one randomly selected occurrence point per grid was retained for model analysis (Dai et al. 2019). After filtering, the final dataset included 226 occurrence points for brown bears, 116 for marmots, and 131 for pikas.

### 2.3 Collection of Bio‐ Variables and Environmental Data

To assess habitat suitability under both current and future climatic conditions, we incorporated multiple environmental variables, including bioclimatic, Land Use/Cover Change (LUCC), topographical, and anthropogenic factors (Table 1). The selected bioclimatic variables play a critical role in shaping the distribution of brown bears and their primary prey species. Brown bears, adapted to high‐altitude environments, depend on climate conditions, habitat availability, and food resources, while marmots and pikas, as key prey species, inhabit similar ecological niches influenced by temperature and precipitation patterns (Dai et al. 2019; Zhou et al. 2021). Given the unavailability of projected future Digital Elevation Model (DEM) and Human Influence Index (HII) data, we followed previous studies (Stanton et al. 2012) and assumed these variables remained constant over time. HII is a global dataset of 1‐km grid cells, created from nine global data layers covering human population pressure (population density), human land use and infrastructure (built‐up areas, nighttime lights, land use/land cover), and human access (coastlines, roads, railroads, navigable rivers).

**TABLE 1:** Environmental variables used in the study.

Variable category	Description	Resolution	Time period	Source
Bioclimatic Variables	19 temperature and precipitation‐related variables (Bio 1‐Bio 19)	1 km	1950–2000 (current)	WorldClim 1.4 (https://www.worldclim.org/)
	Future climate projections under three GCMs (RCP2.6, RCP4.5, RCP8.5)	1 km	2061–2080 (2070s)	WorldClim 1.4 (Ye et al. 2018; Yu et al. 2019)
LUCC	Land Use/Cover Change	30 m	2010 (current), 2070s (future under RCP2.6, RCP4.5, RCP8.5)	Finer Resolution Observation and Monitoring‐Global Land Cover (Li et al. 2016)
Elevation	Digital Elevation Model (DEM; ELE)	30 m	Assumed constant	Geospatial Data Cloud (http://www.gscloud.cn/)
Anthropogenic Impact	Human Influence Index (HII)	1 km	Assumed constant	SEDAC (http://sedac.ciesin.columbia.edu/)

To ensure consistency across datasets, all spatial layers were standardized to a common coordinate system (GCS_WGS_1984), resolution (1 km), and spatial extent using ArcGIS 10.8 (ESRI Inc., Redlands, CA, USA). To mitigate multicollinearity, we computed pairwise correlation coefficients using the Band Collection Statistics tool in ArcGIS 10.8. Variables exhibiting high collinearity (|*r*| > 0.7) were excluded from the analysis (Phillips et al. 2006). Following this screening process, eight key environmental variables were retained for modeling the bears' and their prey's current and future distributions. These selected variables included: Mean Diurnal Range (Bio 2), Temperature Constancy (Bio 3), Annual Precipitation (Bio 12), Precipitation Seasonality (Bio 15), Precipitation of Driest Quarter (Bio 17), elevation (ELE), Land Use/Cover Change (LUCC), and Human Influence Index (HII).

### 2.4 Simulation of the Distribution of Brown Bears and Their Primary Prey

The MaxEnt model (Maximum Entropy Model) is a widely used machine learning approach for predicting species habitat suitability, particularly effective when occurrence data are limited or spatially sparse (Phillips et al. 2006; Phillips and Dudík 2008; Merow et al. 2013). It estimates species distribution by identifying the probability distribution that maximizes entropy under given environmental constraints. In ecological studies, MaxEnt is commonly applied to assess suitable habitats based on environmental variables such as climate, topography, LUCC, and human activity. By analyzing environmental conditions at known occurrence records, the model establishes species‐environment relationships and projects potential distributions into unsampled areas (Phillips et al. 2006). Although the use of a single modeling algorithm may introduce certain uncertainties and potentially limit the generalizability of predictions (Abrahms et al. 2019; Norberg et al. 2019), MaxEnt was considered the most appropriate and robust approach for this study given the nature of the data, which consisted of presence‐only records and a limited number of occurrence points (Phillips et al. 2006; Merow et al. 2013; Elith et al. 2011). MaxEnt has been widely applied and validated in ecological studies under similar data constraints, demonstrating reliable predictive performance even with sparse datasets (Phillips et al. 2006; Phillips and Dudík 2008).

Separate MaxEnt models were constructed independently for each species (brown bear, marmot, and pika) to identify species‐specific ecological responses and habitat suitability. For MaxEnt (V3.4.4) model parameterization, we set a random test percentage of 25% and a regularization multiplier of 1. The model was run with 15 replicates, employing cross‐validation to ensure robustness. Performance was evaluated using the area under the receiver operating characteristic curve (AUC), a standard metric where values closer to 1 indicate higher predictive accuracy (Phillips et al. 2006). To classify suitable habitats, we applied the Maximum Training Sensitivity Plus Specificity (MTSPS) threshold, which balances sensitivity and specificity (Dai, Peng, et al. 2021). Grids with probability values exceeding this threshold were designated as suitable habitats.

### 2.5 Habitat Suitability Assessment Under Three Scenarios

To systematically assess the habitat suitability for brown bears, we developed three distinct habitat scenarios based on the spatial distributions of the brown bear and its primary prey species. This selection was informed by previous analyses of brown bear diets and extensive field observations, which consistently indicate that animal prey (particularly marmots and pikas) constitute the majority of the bear's natural food sources in the high‐altitude environment of the Qinghai‐Tibet Plateau (Dai, Hacker, et al. 2021; Nawaz et al. 2019; Wu 2014; Xu et al. 2006). Although brown bears do consume plant material, vegetation represents a comparatively minor component of their diet due to the harsh environmental conditions, including low temperatures, thin air, and sparse vegetation cover, which limit the availability of edible plants (Dai, Hacker, et al. 2021; Xu et al. 2006). Focusing on these two key prey species is therefore ecologically justified, as they provide the bulk of digestible energy required to sustain the bears in this extreme environment. Furthermore, marmots and pikas exhibit relatively predictable spatial distributions compared with scattered plant resources, making them more suitable for modeling habitat use and informing conservation scenarios.

#### 2.5.1 Scenario 1 (Ideal Distribution Range; S1)

This scenario represents the optimal ecological condition where the distribution of brown bear habitats aligns closely with the spatial distribution of their primary prey. In this ideal state, both the brown bear and its prey species coexist within overlapping habitats, providing the most favorable conditions for the survival and reproduction of the brown bear.

#### 2.5.2 Scenario 2 (Stepping Stone; S2)

In this scenario, prey species are present, but suitable habitats for brown bears are absent or fragmented. These areas, although supporting prey populations, are considered “stepping stones” that may facilitate some movement or connectivity but pose challenges for brown bears due to the absence of adequate habitats. This scenario highlights potential ecological gaps that could impede the bears' survival and adaptation in these regions.

#### 2.5.3 Scenario 3 (Potential Human‐Bear Conflict Area; S3)

This scenario encompasses areas predicted to provide suitable habitats for brown bears, but where prey availability or spatial distribution is limited or absent. Such predator–prey mismatches may compel bears to expand their foraging ranges or rely on alternative food sources, thereby increasing their incursions into human‐dominated landscapes. Consequently, these areas may represent potential hotspots for human‐bear conflicts driven by resource scarcity, posing risks to both bear survival and local livelihoods, while potentially altering broader ecosystem dynamics. Similar scenarios have been proposed in previous studies examining climate‐induced shifts in carnivore behavior (Zhang et al. 2025).

By comparing and analyzing these three scenarios, we comprehensively assess the complexity of brown bear habitat suitability and examine the intricate relationship between habitat quality and food resource availability. This approach provides a robust framework for understanding the ecological challenges and opportunities faced by brown bears in a rapidly changing environment.

## 3 Results

### 3.1 Accuracy Performance of Model Results

**FIGURE 2:**
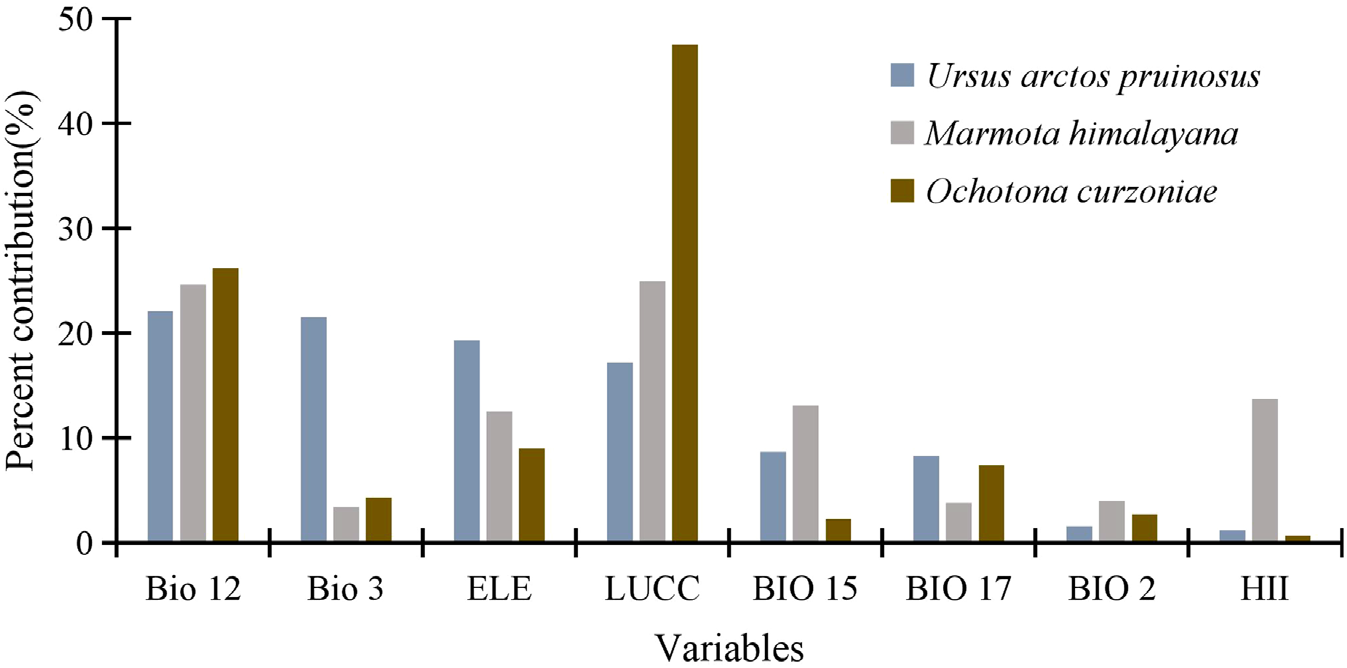
Analysis of variable contributions.

In the MaxEnt modeling results, significant differences were observed in the contribution rates of environmental variables to habitat suitability predictions for the brown bear, marmot, and pika, reflecting species‐specific ecological response mechanisms. The distribution of the brown bear was primarily influenced by a combination of climatic variables (Bio12, Bio3), elevation, and LUCC, with Bio3 contributing 21.5%, which is substantially higher than its contribution to the distributions of the marmot (3.4%) and the pika (4.3%). In contrast, the distributions of the marmot and pika were more strongly driven by LUCC, particularly for the pika, for which LUCC accounted for as much as 47.5% of the predictive power. Additionally, Bio12 emerged as a critical factor across all three species, exerting especially notable influence on the pika (26.2%) and the marmot (24.6%). Notably, the marmot exhibited the highest sensitivity to human influence (HII), with a contribution rate of 13.7%, whereas the brown bear (1.2%) and the pika (0.7%) were comparatively less affected (Figure 2).

### 3.2 Projected Distributions of Three Species

**FIGURE 3:**
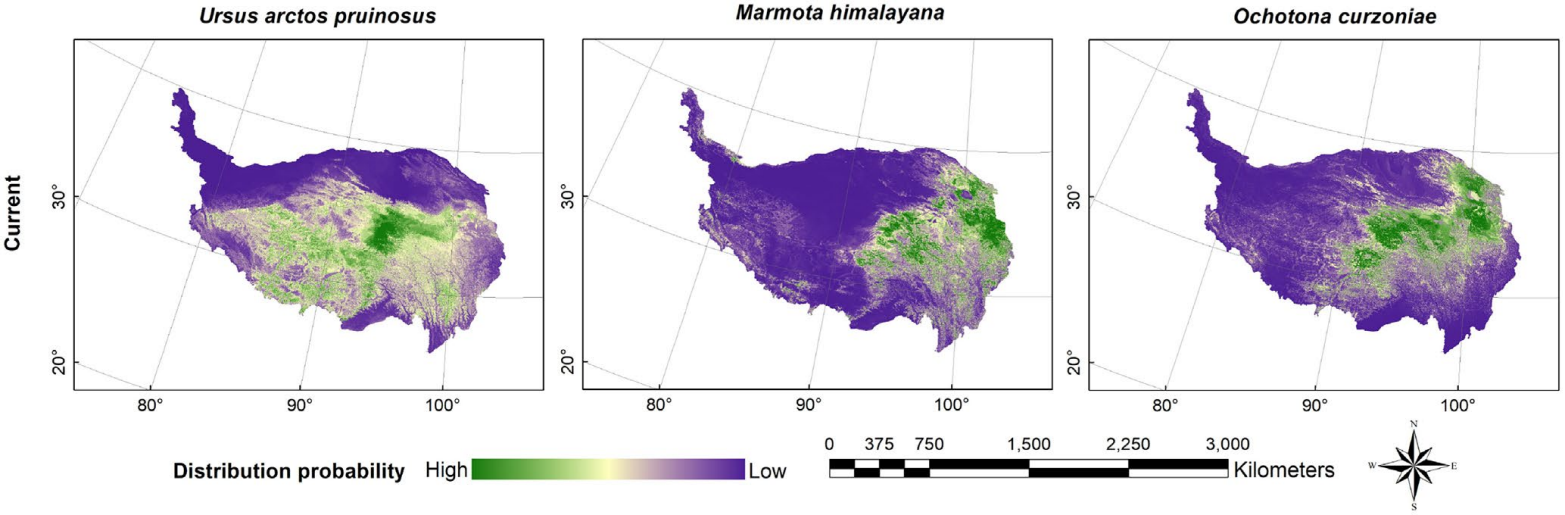
The current distribution probability of Tibetan brown bear ( *Ursus arctos pruinosus* ), marmot ( *Marmota himalayana* ), and pika ( *Ochotona curzoniae* ) on the QTP.

**FIGURE 4:**
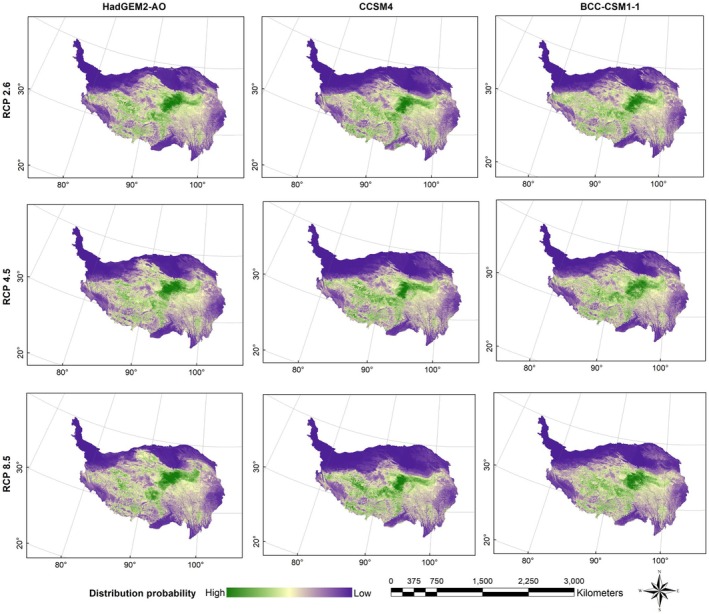
The distribution probability of Tibetan brown bear ( *Ursus arctos pruinosus* ) under future (scenarios of RCP2.6, RCP4.5, and RCP8.5 in the 2070s) climate and land use change scenarios on the Qinghai‐Tibet Plateau.

**FIGURE 5:**
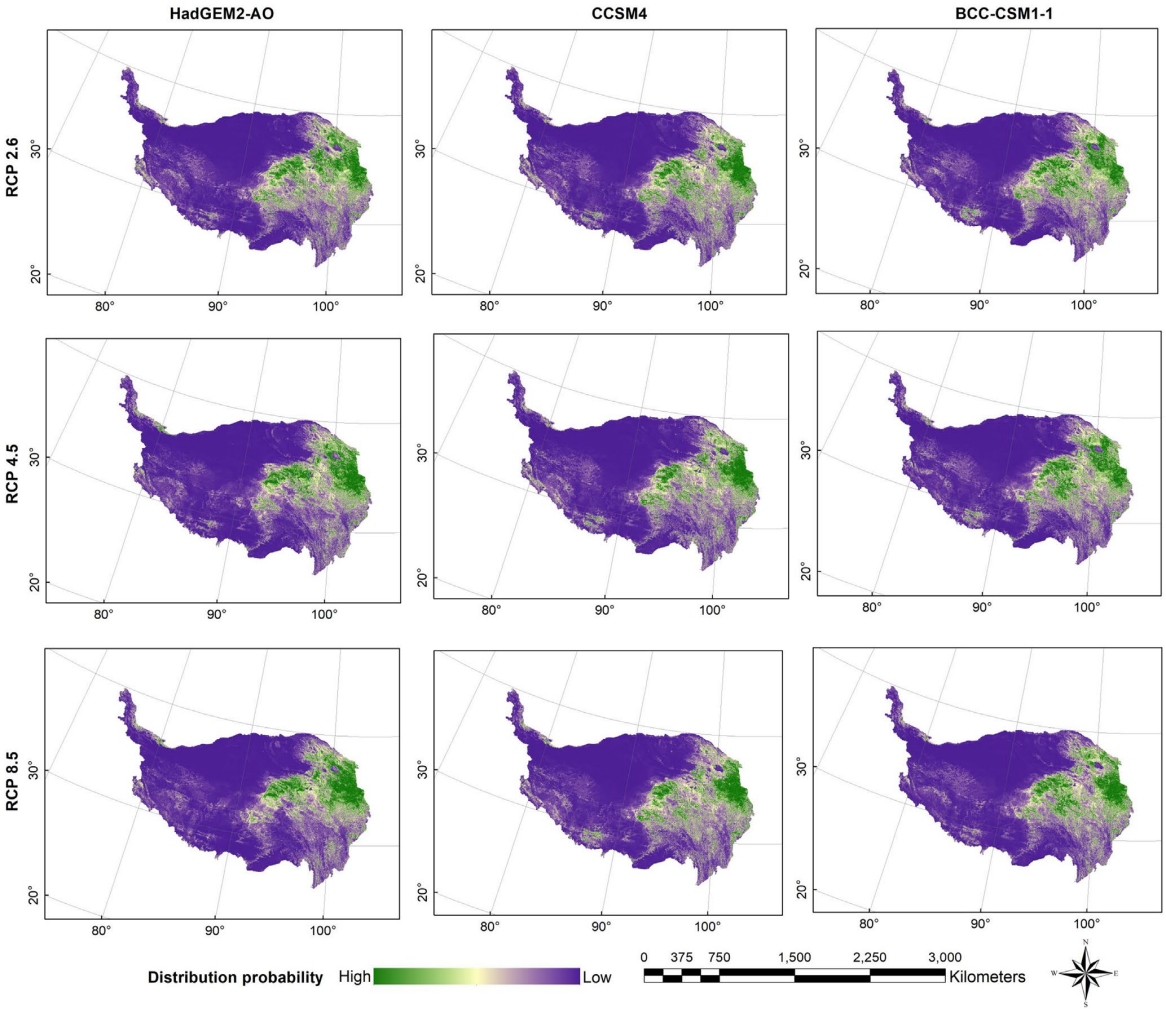
The distribution probability of marmot ( *Marmota himalayana* ) under future (scenarios of RCP2.6, RCP4.5, and RCP8.5 in the 2070s) climate and land use change scenarios on the Qinghai‐Tibet Plateau.

**FIGURE 6:**
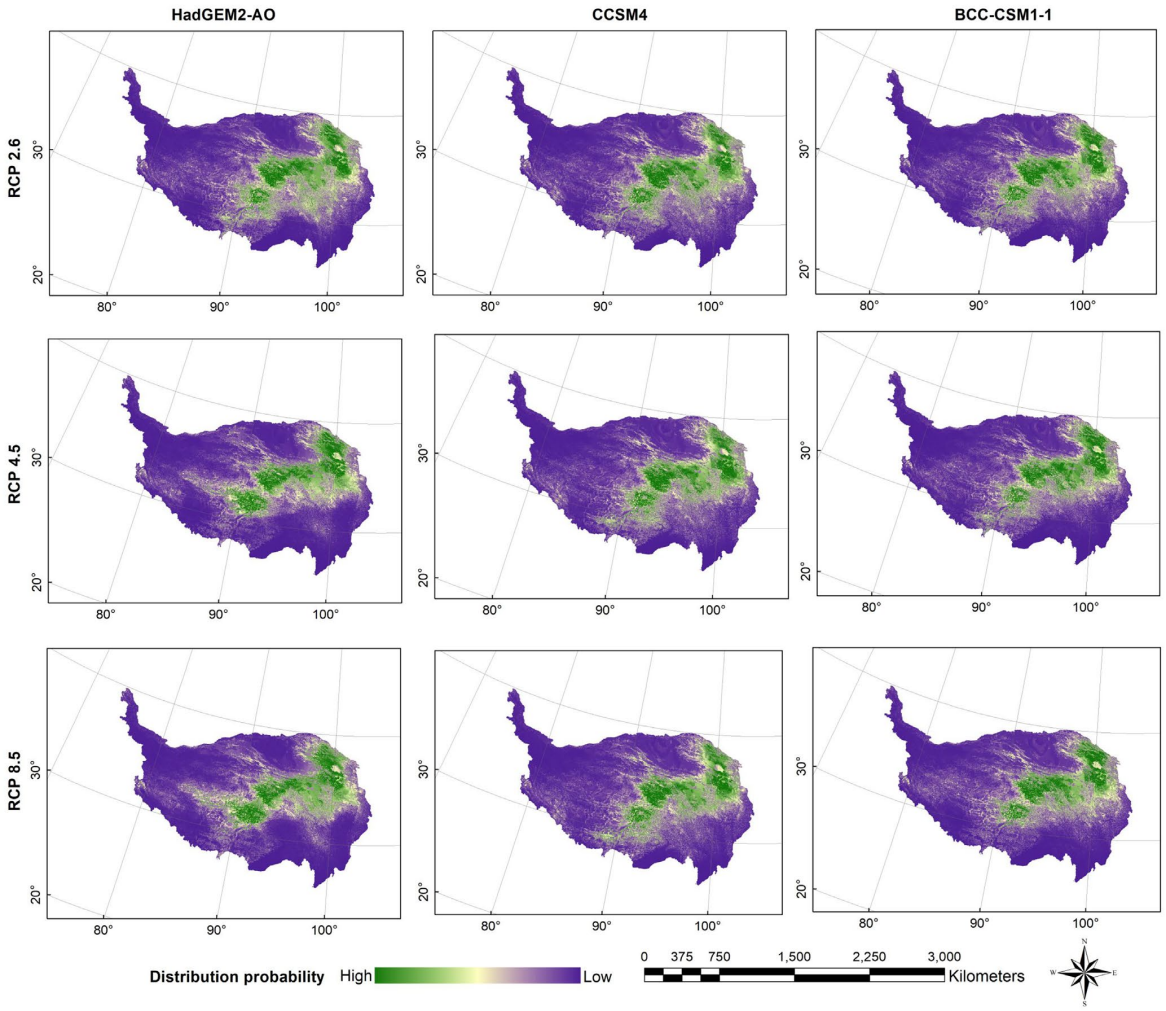
The distribution probability of pika ( *Ochotona curzoniae* ) under future (scenarios of RCP2.6, RCP4.5, and RCP8.5 in the 2070s) climate and land use change scenarios on the Qinghai‐Tibet Plateau.

**FIGURE 7:**
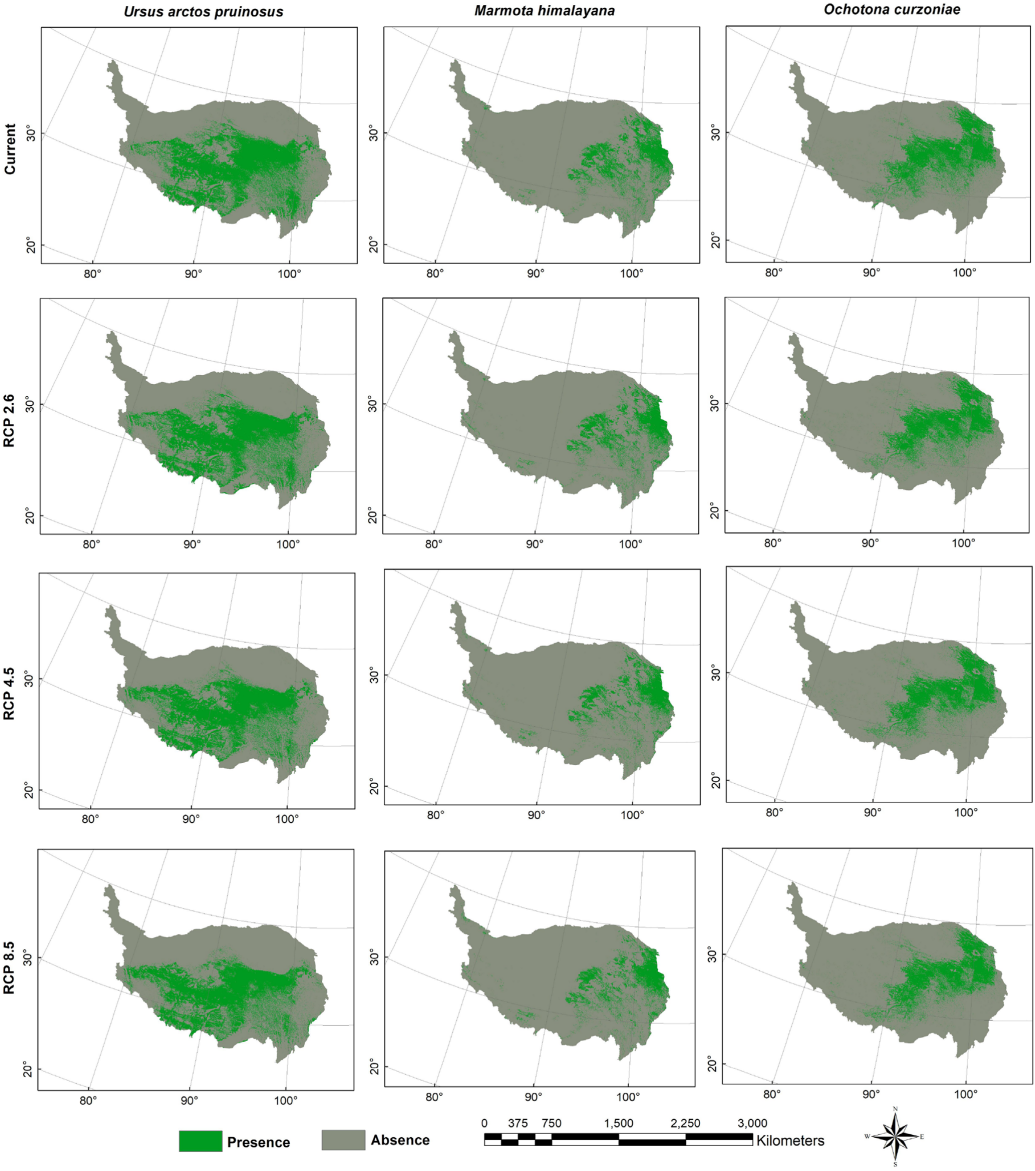
The binary distribution maps of Tibetan brown bear ( *Ursus arctos pruinosus* ), marmot ( *Marmota himalayana* ), and pika ( *Ochotona curzoniae* ) under the current and future (scenarios of RCP2.6, RCP4.5 and RCP8.5 in 2070) climate and land use change scenarios on the Qinghai‐Tibet Plateau. Future distributions are the intersection area of the three GCMs (HadGEM2‐AO, CCSM4 and BCC‐CSM1‐1).

Figures 3, 4, 5, 6 presented the probability distributions of brown bears, marmots, and pikas under current and future climate scenarios and land use changes. The average logistic threshold values of MTSPS for brown bears, marmots, and pikas were 0.3265, 0.3552, and 0.3332, respectively. Binary distribution maps for the three species were generated based on these MTSPS values (Figure 7).

Under current climatic conditions, brown bears are primarily distributed across the Xizang Autonomous Region and Qinghai Province (Figure 7). Model predictions indicated that under future climate scenarios, the suitable habitat for brown bears will undergo pronounced spatial restructuring, with a marked concentration toward the Xizang Autonomous Region. Particularly under the extreme RCP8.5 scenario, the proportion of suitable habitat in the Xizang Autonomous Region is projected to increase to 60.64%, establishing it as the core distribution area (Table 2). In contrast, habitat suitability in Qinghai and Sichuan provinces is expected to decline, while marginal regions such as Yunnan, Xinjiang, and Gansu provinces are predicted to retain only small fragments of habitat, with either minimal changes or an overall decreasing trend. The overall distribution pattern is thus anticipated to shift from a “multi‐regional coexistence” structure toward a “Xizang‐centered dominance,” reflecting a typical climate‐driven contraction of ecological niches and a plateau aggregation phenomenon.

**TABLE 2:** The potential distribution area of Tibetan brown bear ( *Ursus arctos pruinosus* ) and its primary prey species, the marmot ( *Marmota himalayana* ) and pika ( *Ochotona curzoniae* ), under current and future climate change scenarios on the Qinghai‐Tibet Plateau.

Species	Administrative region	Current		RCP2.6		RCP4.5		RCP8.5	
		Area (km^2^)	Proportion (%)	Area (km^2^)	Proportion (%)	Area (km^2^)	Proportion (%)	Area (km^2^)	Proportion (%)
*Ursus arctos pruinosus*	Xizang	642,343.61	54.15	651,577.48	56.50	567,331.63	57.48	645,814.36	60.64
	Qinghai	421,295.48	35.52	395,380.60	34.28	346,558.75	35.11	346,268.03	32.51
	Sichuan	102,907.05	8.68	88,633.88	7.69	58,023.57	5.88	64,609.53	6.07
	Yunnan	7533.52	0.64	7177.22	0.62	3160.95	0.32	4136.99	0.39
	Xinjiang	6957.97	0.59	6767.15	0.59	8475.66	0.86	1288.42	0.12
	Gansu	5124.31	0.43	3682.63	0.32	3537.42	0.36	2965.59	0.28
*Marmota himalayana*	Xizang	52,413.97	11.88	49,080.28	11.89	43,156.33	11.42	51,535.47	13.64
	Qinghai	259,639.59	58.86	242,533.23	58.77	227,969.42	60.32	218,038.43	57.72
	Sichuan	78,665.32	17.83	71,804.75	17.40	56,748.35	15.01	57,033.27	15.10
	Yunnan	3823.42	0.87	3224.75	0.78	2903.24	0.77	2297.26	0.61
	Xinjiang	3257.27	0.74	2005.21	0.49	2157.15	0.57	3950.64	1.05
	Gansu	43,289.90	9.81	44,047.56	10.67	45,019.70	11.91	44,906.17	11.89
*Ochotona curzoniae*	Xizang	144,816.69	20.47	134,413.59	19.82	129,228.87	19.59	125,658.35	19.67
	Qinghai	475,453.75	67.20	468,369.41	69.07	460,831.66	69.86	452,042.43	70.75
	Sichuan	45,074.20	6.37	36,371.21	5.36	29,995.39	4.55	25,029.01	3.92
	Yunnan	40.33	0.01	32.61	0.00	14.54	0.00	4.28	0.00
	Xinjiang	3348.85	0.47	2664.37	0.39	3127.92	0.47	3695.65	0.58
	Gansu	38,800.99	5.48	36,285.71	5.35	36,475.27	5.53	32,512.86	5.09

In contrast, the marmots exhibited relatively stable habitat dynamics. Qinghai Province remained the core distribution area for this species under both current and future climate scenarios, consistently maintaining the highest level of habitat suitability across all projections. Although its overall distribution range experienced only minor fluctuations with climate change, a gradual northwestward shift in habitat was observed. This trend was particularly evident under the RCP4.5 and RCP8.5 scenarios, where habitat suitability notably increased in Gansu Province and Xinjiang Uygur Autonomous Region. Conversely, a decline in suitable habitat area in Sichuan Province was recorded across all scenarios (Figure 7).

The habitat distribution of pikas exhibited a pronounced pattern of concentration, with Qinghai Province and the Xizang Autonomous Region currently constituting the absolute core areas. Under future climate change scenarios, this trend was projected to intensify, particularly in Qinghai Province, where both the suitable area and its proportion were predicted to continue increasing, maintaining its dominance across all scenarios. Under the RCP8.5 scenario, suitable habitat areas in peripheral regions such as Sichuan and Yunnan provinces were projected to decrease significantly, by 44.47% and 89.39%, respectively (Table 2). The distribution centroid was expected to shift progressively toward the interior of the QTP, with mounting ecological pressure at the margins. Overall, the habitat structure exhibited a trend toward single‐core centralization, reflecting a typical pattern of marginal contraction and core aggregation in response to climate change (Figure 7).

### 3.3 Spatiotemporal Variation Characteristics of Brown Bear Habitat Under Three Scenarios

**FIGURE 8:**
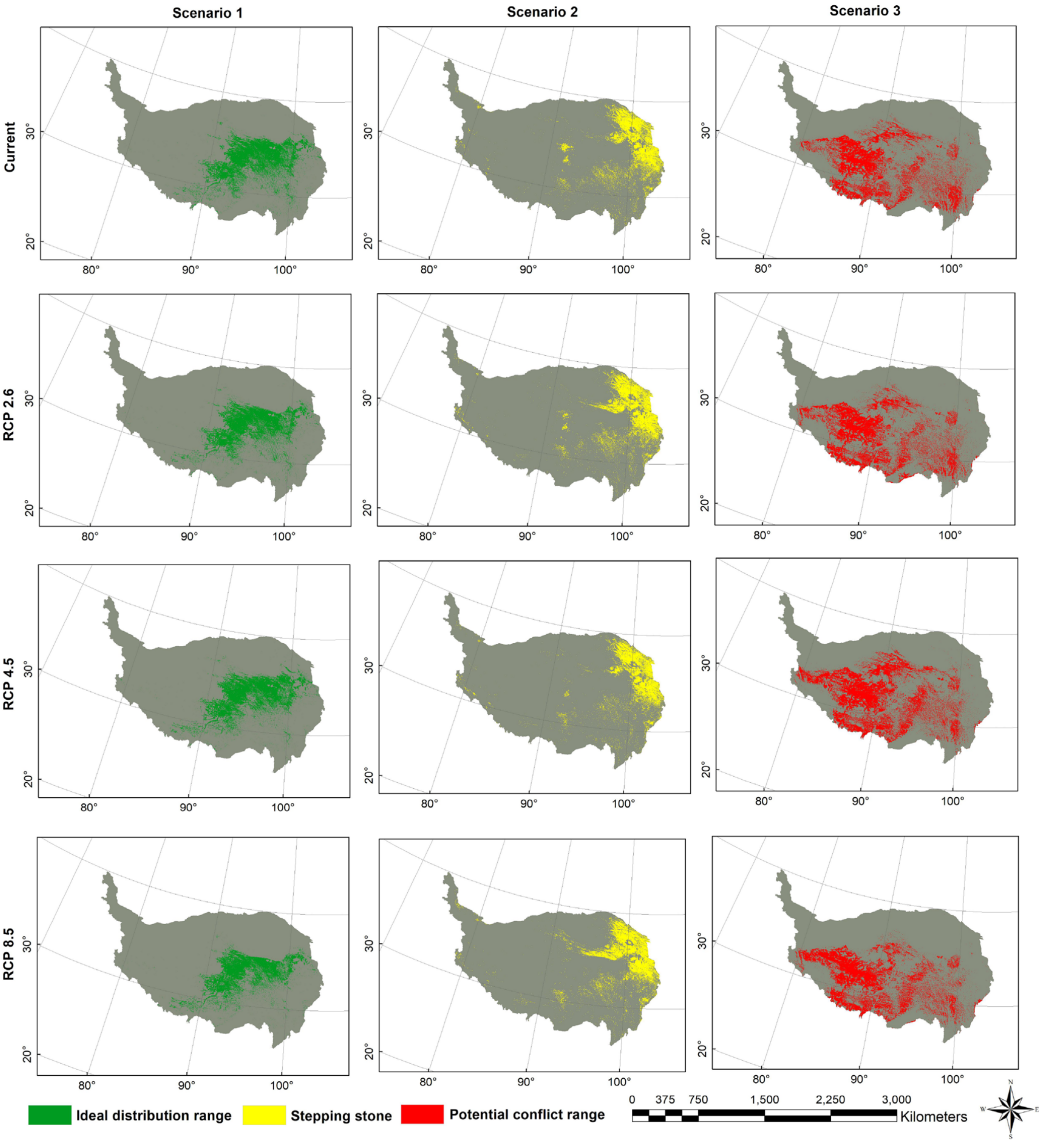
The three habitat scenarios (S1: Ideal distribution range; S2: Stepping stone; S3: Potential human‐bear conflict area) of Tibetan brown bear ( *Ursus arctos pruinosus* ) on the Qinghai‐Tibet Plateau.

A comparative analysis of the spatial distribution patterns of brown bear habitat types on the QTP under current and future climate change scenarios (RCP2.6, RCP4.5, and RCP8.5) was conducted. The results showed that the area of the ideal distribution range (S1) was projected to decline under all future scenarios, with the most pronounced decrease occurring under the RCP8.5 scenario, where the area shrank from 672,069.89 to 524,734.89 km^2^, representing a 21.92% reduction. The area of the stepping stone (S2) exhibited fluctuations across scenarios, with the lowest extent observed under RCP4.5 (415,412.91 km^2^) (Figure 8, Table 3). In contrast, the potential conflict area (S3) generally increased, particularly under RCP4.5, expanding to 787,833.15 km^2^, which represented a 17.03% rise compared to the current extent (Figure 8, Table 3).

**TABLE 3:** The statistics of three habitat scenarios (S1: Ideal distribution range; S2: Stepping stone; S3: Potential conflict area) of Tibetan brown bear ( *Ursus arctos pruinosus* ) on the Qinghai‐Tibet Plateau.

Time	S1		S2		S3	
	Area/km^2^	Percentage/%	Area/km^2^	Percentage/%	Area/km^2^	Percentage/%
Current	672,069.89	27.70	476,582.59	25.52	673,196.01	24.03
Future (2070s)						
RCP2.6	607,325.00	25.03	483,532.11	25.89	686,836.28	24.52
RCP4.5	622,232.59	25.64	415,412.91	22.24	787,833.15	28.12
RCP8.5	524,734.89	21.63	491,984.19	26.34	653,517.54	23.33

## 4 Discussion

Species exhibit distinct ecological responses to environmental drivers (Bradie and Leung 2017; Fei et al. 2017). The brown bear shows a strong dependence on climatic variables, particularly temperature constancy, likely reflecting its thermoregulatory demands and hibernation requirements (Evans et al. 2016). In high‐altitude environments, marked temperature variability may constrain its activity range and access to food resources, thereby affecting population dynamics. This reliance on grassland ecosystems is not unique to Tibetan brown bears, but is also evident in Himalayan brown bear ( *Ursus arctos isabellinus* ) populations, reflecting a shared ecological trait among brown bears inhabiting high‐altitude and steppe environments (Nawaz et al. 2019). In contrast, the distribution of the pika is primarily shaped by LUCC, indicating high sensitivity to habitat structural alterations. Given its reliance on grassland ecosystems, land degradation and agricultural expansion can substantially reduce suitable foraging and breeding habitats. The marmot is mainly influenced by annual precipitation and human influence index, suggesting a dual vulnerability to changing climatic regimes and anthropogenic disturbances such as overgrazing and infrastructure development. Our findings underscore the species‐specific pathways through which climate and land use change interact to shape species distributions in alpine grassland ecosystems. The brown bear's strong dependence on relatively stable climatic conditions, particularly consistent temperature patterns necessary for thermoregulation and hibernation, may increase its vulnerability to future climate variability. In contrast, the habitat specificity of the pika and marmot highlights the biodiversity risks posed by continued land use transformation.

Based on habitat suitability projections under multiple climate scenarios, our study reveals distinct response patterns of the brown bear and its primary natural food sources, highlighting a trend of reshaping species distributions in alpine ecosystems under global warming. The brown bear, in particular, exhibits a contractional distribution pattern, indicating high sensitivity to climatic changes. Under the high‐emission scenario (RCP8.5), its suitable habitat is projected to become increasingly concentrated in the high‐altitude regions of QTP, while suitable areas in Qinghai and Sichuan provinces are expected to contract, with marginal zones experiencing substantially reduced habitat suitability. This spatial trend suggests that the brown bear possesses limited adaptive capacity to climatic shifts in lower‐altitude peripheral areas, with its distribution likely following a “core persistence, peripheral retreat” dynamic. Such ecological niche contraction underscores the importance of conservation strategies that prioritize the protection and connectivity of high‐altitude core habitats in future high‐temperature scenarios (Heller et al. 2015).

In contrast, the marmot exhibits a relatively stable yet low‐mobility distribution pattern, reflecting an ecological adaptation strategy characterized by resilience but limited migration capacity (Wang et al. 2023). Although its overall suitable habitat range shows little fluctuation, a noticeable northwestward shift in the habitat centroid is observed, suggesting a gradual redistribution of suitable areas. Qinghai Province remains the core suitable region, while the proportions of suitable habitats in Gansu and Xinjiang are projected to increase under future climate scenarios. This indicates a certain degree of ecological resilience, enabling the species to maintain stable adaptability in arid and semi‐arid environments. However, the consistently declining habitat suitability in Sichuan across all scenarios implies that the region may progressively become unsuitable for the species' survival. Therefore, future conservation efforts should prioritize the development of ecological corridors and migration pathways among Qinghai, Gansu, and Xinjiang to enhance the species' spatial adaptability and ensure long‐term persistence.

The pika exhibits a core‐area aggregation response pattern. Its suitable habitat is predominantly concentrated in Qinghai Province and Xizang Autonomous Region, with future climate scenarios projecting an increasing reliance on the Qinghai Province. In contrast, marginal areas such as Sichuan and Yunnan provinces are expected to experience significant habitat contraction, reflecting the species' high sensitivity to climate change and a marked narrowing of its ecological niche. This pattern suggests an intensifying dependence on core alpine regions under global warming, with peripheral populations potentially facing a heightened risk of local extinction. In addition to its ecological sensitivity, the pika is also a highly managed species across the Qinghai‐Tibet Plateau. Long‐term control programs, primarily through poisoning campaigns, have been conducted since the late 1950s to mitigate perceived rangeland degradation. However, these interventions have shown limited long‐term success, as high reproductive rates allow pika populations to recover rapidly (Pech et al. 2007). Moreover, population dynamics are strongly influenced by grazing regimes and habitat management practices. In fenced or lightly grazed grasslands, pika densities tend to remain higher during winter compared to open‐grazed pastures, indicating that vegetation availability and pasture management can mediate overwinter survival (Pech et al. 2007). Therefore, rather than relying solely on chemical control, future management should integrate ecological and socioeconomic considerations, promoting adaptive strategies that balance grassland productivity and biodiversity conservation. Ecologically based pest management, including habitat regulation, grazing optimization, and restoration of natural predators, could provide more sustainable outcomes for both rangeland health and pika population stability.

Future climate change is expected to profoundly affect the spatial distribution of suitable habitats for the brown bear, posing new challenges for its population persistence and conservation management (Su et al. 2018). The continued reduction in optimal habitat area may constrain access to essential resources such as food, water, and breeding sites, thereby undermining population stability and dispersal capacity. Based on our species distribution projections, suitable habitats for brown bears may increasingly overlap with lower‐altitude areas where human settlements exist. While our study does not directly analyze human‐bear conflict incidents, this spatial overlap highlights a potential risk of human‐wildlife interactions that warrants further investigation (Khosravi et al. 2023). Accordingly, future research could focus on assessing these potential interactions and designing evidence‐based management strategies. Such studies may consider monitoring bear movements in peripheral areas, evaluating habitat‐use patterns relative to human activity, and exploring adaptive conservation measures to enhance coexistence.

To mitigate the far‐reaching impacts of future climate change on the habitat patterns of the brown bear and its key prey species, an integrated conservation strategy should be developed with a core focus on core habitat protection, connectivity enhancement, and conflict mitigation. First, it is essential to strengthen the protection of high‐suitability core habitats, particularly in regions such as Xizang Autonomous Region. This can be achieved through the delineation of ecological protection redlines (strictly protected zones designated to safeguard key ecosystem functions and maintain ecological security), strict restrictions on infrastructure development, and the establishment or expansion of protected areas to preserve ecologically stable high‐altitude habitats. Second, in response to the projected spatial shifts in marmot habitat, a well‐designed ecological corridor network should be established between Qinghai, Gansu, and Xinjiang. Conservation actions such as grassland restoration, optimization of LUCC patterns, and the retirement of grazing lands are recommended to enhance habitat connectivity and facilitate species dispersal. For the pika, conservation efforts should focus on maintaining the ecological integrity of its core habitats in Qinghai and promoting sustainable grassland management to prevent habitat degradation caused by overexploitation and land‐use intensification.

In response to the projected expansion of potential human‐bear conflict zones, a more systematic early warning and mitigation framework should be implemented in the peripheral areas of the QTP. First, a comprehensive monitoring network for brown bear activity should be established in high‐risk areas, utilizing infrared cameras, GPS collars, and drone patrols to enable real‐time tracking of bear movements. Concurrently, a spatiotemporal risk assessment model should be developed by integrating climate variables, seasonal dynamics, and fluctuations in food resources, with the aim of delivering timely early‐warning information to herders and local communities. Second, the installation of wildlife‐friendly barriers, such as solar‐powered electric fences and reinforced metal enclosures, should be promoted to prevent bears from breaking into houses and ensure human safety in high‐risk areas. In parallel, community‐based compensation and insurance schemes mainly covering house damage and livestock loss should be strengthened by simplifying claims procedures, enhancing response speed and compensation standards, and thereby increasing public support for conservation initiatives. Additionally, education and capacity‐building programs should be implemented to raise awareness of human‐bear coexistence, including training on bear behavior, use of acoustic deterrents, and dissemination of safety knowledge. Lastly, local community participation in co‐management frameworks should be actively encouraged to establish a long‐term collaborative governance mechanism involving conservation authorities, local governments, and pastoralist communities, which will ultimately advance both ecological conservation and human safety objectives.

## 5 Conclusion

Our study systematically assessed the differential responses of the Tibetan brown bear and its primary prey species to climate change and anthropogenic disturbances on the QTP. The findings reveal that each species exhibits distinct ecological preferences and vulnerability patterns: the brown bear shows a marked dependence on temperature constancy and demonstrates a contraction‐type distribution shift under high‐emission scenarios; the marmot displays spatial migration tendencies with relatively stable habitat suitability, suggesting stronger ecological resilience; and the pika demonstrates pronounced habitat sensitivity with significant niche contraction toward core areas. These divergent response mechanisms not only reflect the complexity of species‐environment interactions in alpine ecosystems but also highlight the need for differentiated conservation strategies. Moreover, the study emphasizes the potential threats of climate‐induced habitat loss, fragmentation, and intensified human‐wildlife conflicts, underscoring the urgent need for adaptive management frameworks in high‐altitude grassland systems.

## Author Contributions

**Qiaoyun Sun:** conceptualization (lead), methodology (equal), writing – original draft (lead). **Kunyuan Wanghe:** investigation (equal), writing – review and editing (equal). **Yunchuan Dai:** conceptualization (lead), funding acquisition (lead), investigation (equal), writing – original draft (equal).

## Funding

This work was supported by the 2023 Award Fund of Qinghai Provincial Key Laboratory of Animal Ecological Genomics (Grant No. QHEG‐2024‐09).

## Conflicts of Interest

The authors declare no conflicts of interest.

## Data Availability

All data used in this study are publicly available. Species occurrence data for the three species have been archived in the Dryad Digital Repository and are accessible at https://doi.org/10.5061/dryad.9zw3r22tm. Environmental variables were obtained from open‐access sources including WorldClim v2.1 (https://www.worldclim.org/data/index.html), ASTER GDEM V2 (https://www.gscloud.cn/), and Last of the Wild v2 (https://sedac.ciesin.columbia.edu/data/set/wildareas‐v2‐last‐of‐the‐wild‐geographic). The data were formatted according to the minimum standards outlined in Ecology and Evolution's data policy.
